# Addressing Inequalities in Long Covid Healthcare: A Mixed‐Methods Study on Building Inclusive Services

**DOI:** 10.1111/hex.70336

**Published:** 2025-07-02

**Authors:** Ghazala Mir, Jordan Mullard, Amy Parkin, Cassie Lee, Jonathan Clarke, Johannes H. De Kock, Denys Prociuk, Julie L. Darbyshire, Sophie Evans, Manoj Sivan, LOCOMOTION Consortium

**Affiliations:** ^1^ Leeds Institute of Health Sciences University of Leeds Leeds UK; ^2^ Population Health Sciences Institute, Newcastle University, Newcastle, UK; Gateshead HDRC, Gateshead Council, Gateshead, UK; University of Johannesburg Johannesburg South Africa; ^3^ Academic Department of Rehabilitation Medicine University of Leeds Leeds UK; ^4^ National Demonstration Centre in Rehabilitation, Leeds Teaching Hospitals NHS Trust Leeds UK; ^5^ Imperial College Healthcare NHS Trust London UK; ^6^ Department of Mathematics Imperial College London London UK; ^7^ NHS Highland; COVID recovery Service Inverness UK; ^8^ Community Psychosocial Research (COMPRES) North West University Potchefstroom South Africa; ^9^ Department of Computing Imperial College London London UK; ^10^ Nuffield Department of Primary Care Health Sciences University of Oxford Oxford UK; ^11^ LOCOMOTION Long Covid Patient Advisory Group member Leeds UK; ^12^ Institute of Rheumatology & Musculoskeletal Medicine University of Leeds Leeds UK; ^13^ https://locomotion.leeds.ac.uk/wp-content/uploads/sites/74/2024/05/LOCOMOTION-Consortium-List.pdf

**Keywords:** care pathways, general practice, inequalities, long Covid

## Abstract

**Background:**

Long Covid (LC) significantly impacts health, economic and social activities. Women, deprived, learning disability, homeless and some minority ethnic populations have high prevalence rates but low access to support, indicating health inequities in LC care.

**Aim:**

To identify health inequities in LC care and inclusion strategies that align with the priorities of people with LC.

**Design and Setting:**

Mixed‐methods study employing qualitative data from people with LC, professional experts, LC clinic staff and primary care data from North West (NW) London GPs.

**Method:**

Qualitative interviews with 23 people with LC and 18 professional experts explored the experience of diagnosis and support for people from disadvantaged groups. Framework analysis identified themes that informed the subsequent collection of clinic and primary care data. Staff from 10 LC clinics across England provided survey and qualitative data describing their initiatives to identify and reduce inequalities. Descriptive quantitative analysis of NW London adult primary care records (*n* = 6078), linked to hospital use across England, explored LC diagnosis and care pathways for diverse groups of people with LC.

**Results:**

Study participants from disadvantaged groups reported delays in formal diagnosis and specialist referrals being initated and had low trust in healthcare services. They described difficulties in obtaining information, advice and support as barriers to access specialist referrals. LC clinics confirmed the under‐referral of those from the most disadvantaged groups compared to the general population. Primary care data confirmed under‐referral of people with LC to specialist clinics; however, incomplete data across the LC clinical pathway prevented an analysis of equity in referral patterns between population groups.

Clinics used various strategies to improve access and increase the flow of disadvantaged groups from primary care to LC services. Interventions included data collection to identify underserved groups, targeting outreach to primary care and community providers, adapting clinic provision and developing care pathways involving multidisciplinary teams (MDTs), primary and secondary care practitioners and third sector organisations. These activities were not widespread, however, and were particular to a few clinics.

**Conclusion:**

Strategies to improve access to LC healthcare provide a starting point to explore inclusive care pathways for people with LC from disadvantaged social groups. Future research should focus on the effectiveness of initiatives to increase access to specialist LC provision among disadvantaged groups and establish greater trust in healthcare providers.

**Priorities of People With LC for Healthcare Practice:**

This study highlights the need for health system practitioners to identify under‐represented groups and target inclusion initiatives at these populations in sensitive and appropriate ways. Improved diagnosis and support for such populations would be helped by training health and social care practitioners to recognise and accept the accounts of people with LC about their symptoms. Protocols that support consistent practice in referrals for specialist care are also needed. People with LC from disadvantaged groups often lack access to evidence‐based sources of advice and information. Practitioners should provide information on LC while individuals are waiting to receive specialist care and should advocate for support from employers, including work modifications.

**Patient and Public Contribution:**

People with lived experience of LC were involved in the study as members of the research team and LOCOMOTION Patient Advisory Group (PAG). The PAG was involved in the wider study design, including the initial grant application, attending proposal planning meetings and helping to develop the research aim, objectives and questions. During the course of the study, the PAG met quarterly with each other and at least monthly with other research team members to review progress and feed into data collection and analysis processes. PAG members also attended a Quality Improvement Collaborative meeting involving academics and LC practitioners, which discussed initial findings from data analysis of qualitative interviews on LC inequalities. Through these meetings, the group supported and oversaw the study as a whole in terms of how data was collected, recruitment of participants, involvement in data analysis and interpretation, as well as providing more specific advice to all the individual workstreams within the study. A PPI facilitator within the study team provided training and support to PAG members in these areas and was available to respond to other needs expressed by the group. PAG members have also been involved in organising and contributing to a wide range of study dissemination events. PAG involvement throughout the study has ensured that the research is aligned with the key research priorities of people diagnosed with LC as well as those with LC symptoms but no formal diagnosis. PAG members were recruited through and linked to the LC clinics involved in the study and have helped disseminate study findings to local clinical practice, the lay public and other LC centres with which they are involved. S.E. is a PAG member from a minority ethnic background and a co‐author on the paper. She has been involved in overseeing and supporting data collection and interpretation relating to inequalities affecting people with LC and has contributed to the preparation of this manuscript from an early draft to production of the final version.

## 1 Introduction

Long Covid (LC) is defined as persistent COVID‐19 symptoms more than 4 weeks after acute infection and is a growing global problem [1]. Commonly reported symptoms can adversely affect day‐to‐day activities significantly [2] and include fatigue, cognitive dysfunction, shortness of breath, pain and mood problems [3, 4]. LC also impacts economic, social and caring activities [5].

LC affects around 200 million people worldwide [6], including almost 2 million in the United Kingdom [3]. A recent meta‐analysis indicated that 45% of COVID‐19 survivors experienced unresolved symptoms at around 4 months [7]. Among working‐age people, the likelihood of economic inactivity after a confirmed COVID‐19 infection was significantly higher than before infection [8, 9].

Large studies on the prevalence of LC show higher rates in economically deprived, learning disability, homeless and some minority ethnic populations [4, 10], who are also more likely to have underlying health conditions and experience social isolation [11]. A significant body of evidence links health inequalities in these groups to reduced access to support or involvement in decision‐making about their health and poorer quality healthcare compared to the general population [12, 13, 14]. Similar barriers have been reported by people with LC from such groups, including unresponsive services, inadequate support and mistrust or fear linked to negative interactions with healthcare providers [15, 16, 17].

**Figure 1:**
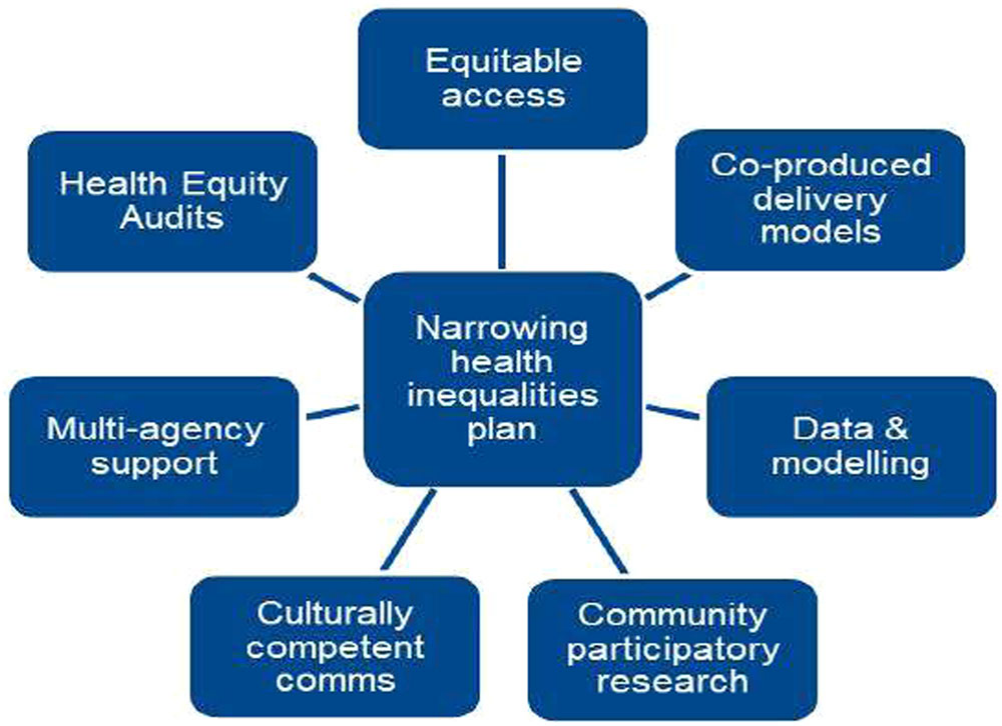
NHS LC Plan for reducing inequality.

Reducing health inequalities for people with LC is a key aim of the National Health Service (NHS) LC Plan 2021/22 [18] which promotes the use of clinical data, engagement with communities, culturally competent services and multiagency collaboration (Figure 1).

We aimed to explore the experience of access to LC healthcare for people from socially excluded populations and identify inclusion strategies that address inequities in LC care. We examined whether these strategies aligned with the priorities of people with LC and identified examples of good practice from clinics within the LOCOMOTION research consortium [19] that could inform current practice and future research.

## 2 Methods

Using a mixed‐methods approach, we explored diverse stakeholder perspectives on inclusive LC support for members of socially marginalised groups. In 2021–3, qualitative interviews were conducted with individuals from across the United Kingdom. In 2023, data were collected from primary care records and LC clinics.

### 2.1 Study Participants and Design

Semi‐structured interviews were used to gather qualitative data from individuals in populations identified as underserved [20]. We focused on people with LC who could recall their COVID‐19 infections and subsequent LC symptoms, aged 18 or over and from a range of disadvantaged populations: people from deprived and minority ethnic backgrounds, women, homeless people and people with disabilities; we did not prioritise specific participants within or between these groups. We purposefully recruited through community organisations, posters in GP surgeries, researchers' personal networks and snowballing, particularly targeting individuals who did not attend LC clinics. Participants received a study information sheet, gave informed consent for interviews and received an involvement fee for participation. Interviews explored experiences of LC and any support received in relation to diagnosis, treatment and management (see Supporting Information^1^). Academic, policy and practice experts in LC and inequalities (‘Key Informants’) were also recruited using purposive sampling. These interviews explored issues relating to healthcare access and other issues relevant to inequalities, such as the impact of multiple forms of disadvantage (see Supporting Information^2^).

NVivo 14 software was used to code data and help develop themes using the framework analysis tools of familiarisation, identifying themes and codes, charting, mapping and interpretation [21]. J.M. and G.M. double coded 10% of transcripts and compared themes and codes to agree on a coding framework. We discussed the themes with our PAG and lived experience advisers, after which J.M. coded the remaining transcripts and charted the coded data. J.M. and G.M. then used data charts to map participant experiences and views, interpret the data as a whole and develop findings. Quotations were selected to highlight common and specific experiences that had implications for LC policy or practice development. All interviews were conducted by J.M.; joint coding and interpretation of data by J.M. and G.M. aimed to ensure rigour in data analysis processes and our final interpretations [ibid].

This analysis informed a survey of 10 LC specialist clinics to identify initiatives for addressing inequalities in terms of demographic data collection and analysis and use of such data (see Supporting Information^3^). A description of how these clinics operated is provided elsewhere [22]. Healthcare staff in these clinics, along with people with lived experience of LC, were involved in a Quality Improvement Collaborative at which findings from our qualitative interviews were discussed [22]. A questionnaire was designed to identify the extent to which clinics were aware of inequalities in access to specialist healthcare, challenges to data collection and examples of inclusion initiatives being undertaken to help reduce inequalities.

Primary care data were obtained from the Whole Systems Integrated Care (WSIC) dataset, which comprises individual records of over 2.7 million patients from GP practices in North West (NW) London. These data were linked, using a unique pseudonymised patient identifier from the NHS number to hospital Secondary Uses Service data containing records of appointments, accident and emergency attendances and inpatient admissions to hospitals across England. Patients were identified as having a recorded diagnosis of LC if their primary care record contained one of four LC Systematized Nomenclature of Medicine (SNOMED) clinical terms between 1 January 2020 and 30 September 2023.

Our qualitative findings also informed the analysis of primary care data, which focused on LC care pathways for diverse populations. Demographic variables (sex, ethnicity and socioeconomic status based on the Index of Multiple Deprivation ranks) were compared between patients with and without an LC diagnosis. Descriptive statistics were generated, and the concordance between a primary care diagnosis of LC, LC clinic referrals and attendance at appointments was evaluated. For each patient, the frequency with which one, two or three of an LC diagnosis, LC clinic referral or LC clinic attendance were present was identified and used to construct a Venn diagram. Data extraction was performed using Microsoft SQL Server Management Studio 2018, while data processing and analysis were conducted in Python (v3.7.9) using the Pandas (v1.3.2) and numpy (v1.19.5) libraries.

Findings from the survey and primary care data were combined with qualitative data findings to identify common themes across the three data sources and explain, confirm or enhance findings from each source [23]. We then drew on this triangulation of findings to suggest potential ways forward for how inequities could be addressed. Oversight was provided by the LOCOMOTION Data Management Group, with additional input from the LOCOMOTION Patient Advisory Group, of which S.E. is a member.

## 3 Results

### 3.1 Qualitative Findings

Qualitative interview data were obtained from 23 people living with LC (PWLC) from underserved groups and 18 academic or healthcare experts in LC and inequalities (Key Informants—KIs). Demographic details of these participants and more details of their experience of diagnosis have been published elsewhere [15]. The recruitment process encountered challenges in engaging homeless individuals. Specialist general practitioners (GPs) and voluntary sector organisations indicated that homeless individuals were less likely to associate LC symptoms with COVID‐19, due in part to symptom overlap with drug and alcohol addiction and a lack of diagnostic testing. Additionally, immediate financial, housing and social concerns were reported to take precedence over participation in research, even though involvement fees were offered to all people with LC.

### 3.2 Barriers to LC Support

People with LC identified several barriers to accessing support for LC, including unresponsive services in which they were not believed or supported by GPs or referred for specialist care. There was also a general deficiency in LC‐related knowledge among both patients and primary care providers, and some people with LC expressed mistrust towards healthcare professionals, based on personal or previous community experiences of discrimination. The lack of diagnosis was a cause of considerable emotional distress for people with LC [15] and further complicated interactions with employers, who often did not acknowledge ‘invisible’ symptoms such as fatigue, pain and cognitive dysfunction [8] (see Box 1: Barriers to LC support).

Box 1 **Perspectives of people with LC (PWLC) on healthcare support.**

*Unresponsive services*

As the GP said, ‘there's no such thing as long Covid’ so!

(PWLC11 Mixed‐Black British Female, self‐employed, low SES, 35–45)

It's very difficult! I feel that I have little faith in speaking to the GP because I'm not getting anywhere since November 2020. It's now May 2022 and I've not got anywhere! I haven't had a referral to the long Covid clinic. I haven't had any treatment plan put in place in terms of my breathing … it's just, it's frustrating!

(PWLC01 South Asian Female, mid‐SES, 25–35 years)

If I didn't work at the hospital, I would have just been in limbo

(PWLC22 Black British Female, mid‐high SES)

I had one [GP] who used to say ‘it doesn't really matter what it is! You just need to rest and work less!’ […] I say ‘well can you write me a letter to say this, so I can give it to work?’ ‘Oh no, no, no. If, if they want it, occupational health can ask’

(LC06 Southern European Female, unemployed following sick leave, mid‐SES, 35–45)

According to our survey and what we see anecdotally in the group, it's still terrible. People are waiting months and months and months to be seen.

(KI/PWLC09, White Female/LC Activist, unemployed, low SES, 45–55 years)

*Lack of information*

I now know that there is support for long Covid. But I haven't been informed of that by either the medical surgery that I go to or by the doctors that I saw in hospital either.

(PWLC05 South Asian Male, retired, mid SES, 65–75 years)

I only knew about the long Covid clinic because my sister had been referred. And she probably only knew about the long Covid clinic from work because she works in the NHS … a lot of people are not aware of Covid clinics. And that's a huge barrier. In my own experience, I find that a GP wouldn't refer you to a long Covid clinic unless you personally asked them to do it.

(PWLC01 South Asian Female, mid SES 25–35 years)

*Fear of discrimination, lack of trust*

There's been a lot of, you know, mistakes … things that have been done! People have been treated in not great ways! I just think, you know, that's what's brought the mistrust really.

(PWLC12 Black British Female, low‐SES, 45–55)

*Inappropriate care pathways*

The medical team have managed me by splitting my symptoms up into their specialities. So, I'm under two specialities: infectious diseases for the ongoing fever, which they can't rectify; and I'm under a neurologist/chronic headache consultant because I have a chronic headache. In correspondence rarely do they say this is long Covid.

(PWLC02 Black British Female, mid‐SES, 25–35 years)

KI participants working in LC healthcare and academia confirmed these dynamics:

‘the attitudes of health professionals affect access as well. So if I see you and pigeonhole you as a woman of a certain age and likely to be more on the functional disease spectrum ‐ and there are people who have these views ‐ then I'm less likely, even if there is a LC clinic, then I'm less likely to refer you to it.[…] we know from the first wave that people from certain ethnic minority backgrounds didn't trust the health system […] in terms of vaccination strategy, in terms of the acute waves of the pandemic. They don't trust what the hospitals are doing, what the government is doing, what the GP is doing. And they're going to be difficult to get to the LC clinic’.

KI06, clinical academic and cardiologist

There's a lot of knowledge that's missing. I think that is probably one of the big drivers about who is self‐reporting […] and who can advocate for it

KI10, Community Health Practitioner

### 3.3 Referral to Specialist Care

Where referrals were made to secondary care, this was often to a range of separate clinics dealing with individual symptoms rather than to specialist care for LC. Various tests might be organised through these referrals; however, if these returned normal results, care would end without an overview of the combination of symptoms that an individual experienced (see Box 1: Inappropriate care pathways).

Ten of our participants with LC, mostly healthcare professionals, did manage to organise a referral for specialist care after considerable persistence, using knowledge of NHS provision from within their own networks. What was clear from people with LC (PWLC), however, was that most respondents did not have the necessary energy or resources for such engagement [15] and were left feeling frustrated and neglected (Box 1 PWLC01).

### 3.4 LC Clinic Data

Feedback from professionals working in specialist LC clinics revealed that there was a lack of demographic data in their records of people with LC. Only two of the ten sites were able to provide details of data collection on age, gender, ethnicity, deprivation, disability or homelessness. At one of these clinics, data on the first three categories and possibly a disability flag were recorded on the main record system, with other data recorded via free text that would be difficult to extract.

This context made it difficult to assess the extent of inequities in access to clinics and the impact of operational policies on disadvantaged groups. Only a minority of clinics, however, appeared to be taking steps to address this issue. In a clinic that did collect comprehensive demographic data, under‐representation of people with LC from deprived, ethnic minority and learning disability groups was noted, along with poor referral of these groups from primary care practitioners. No clinic in our sample had any coded record of people with LC with a learning disability, despite the known higher prevalence in this population [24].

### 3.5 Findings From Primary Care Data

Analysis of the larger dataset from primary care practices in NW London indicated better data collection for most excluded groups. Homeless people were identified using a collection of SNOMED codes that encompass homelessness, rough sleeping and temporary lodging circumstances. People were identified by the presence of any of the related codes in the period of 3 years before LC diagnosis. In total, 35 homeless people with LC were identified (0.75% of all people with LC). Despite the existence of clinical codes for learning disability and the higher prevalence of LC associated with both these groups [3, 24], it was not possible to identify sufficient people to include these groups in our analysis.

The socio‐demographic profile of this overall population and those with a recorded diagnosis of LC is published elsewhere [25]. This earlier analysis found that a diagnosis was more likely to be recorded in primary care for women and people from Asian backgrounds or mixed ethnicity than for people from white backgrounds. Surprisingly, a higher risk of LC was found for those in our sample who lived in the third and fourth most deprived quintiles than for those in the most deprived quintile [25].

**Figure 2:**
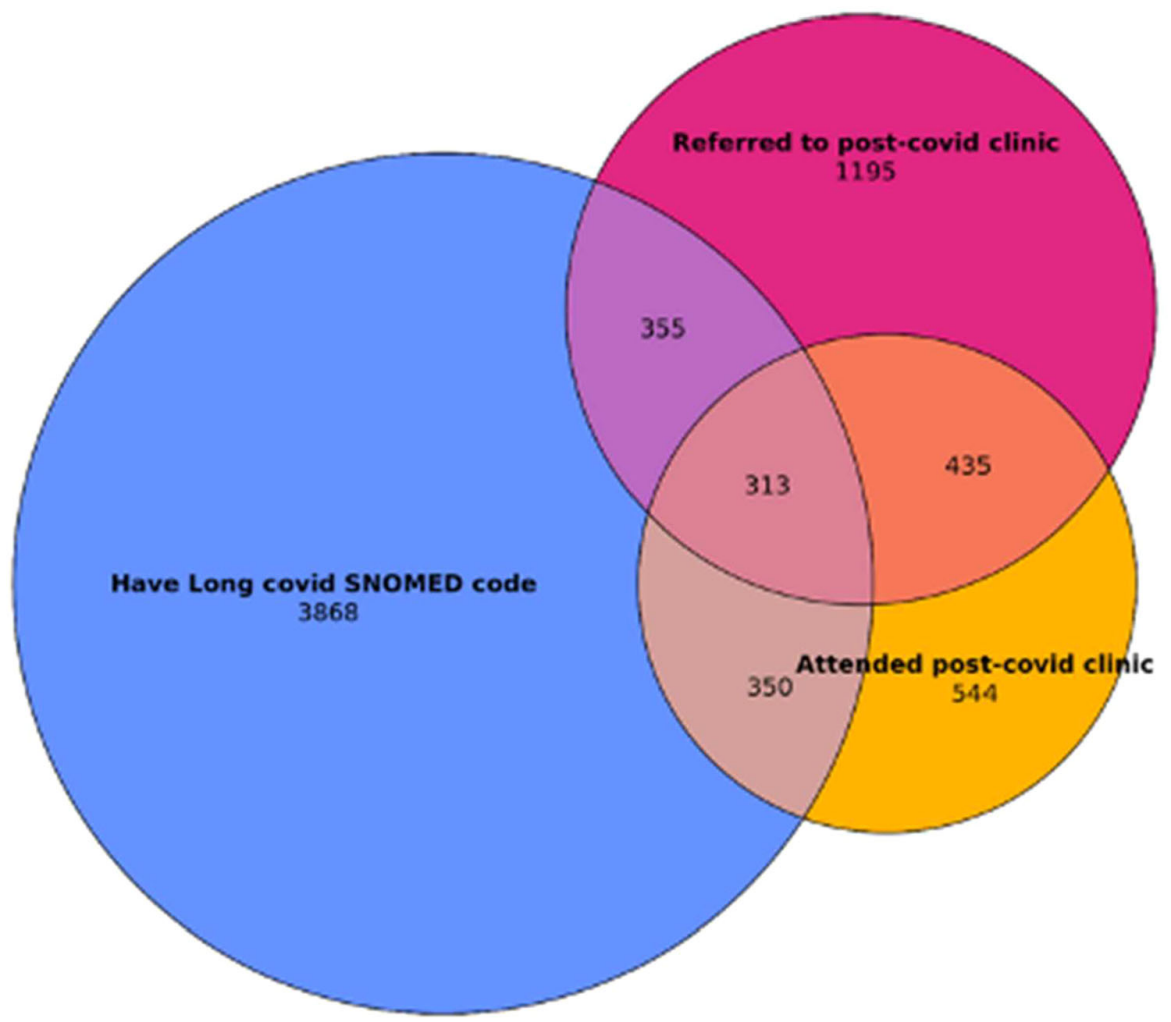
Overlap between recording of LC diagnoses, referral to LC clinics and LC clinic attendance.

Further analysis of the dataset for this paper found low overlap between data on people with an LC diagnosis in primary care, referral to an LC clinic and LC clinic attendance. There were 7060 individuals with a record of either an LC diagnosis, LC clinic referral or LC clinic attendance; however, only 4.4% had records of all three. Over half (54.8%) had only a record of LC in their primary care without a record of LC clinic referral or attendance, while 68.1% of those either referred to or seen in an LC clinic did not have an LC diagnosis in their primary care record. This lack of overlap is visually represented in Figure 2.

While not all people with LC may require onward specialist referral, Figure 2 indicates there is low referral in general for specialist LC care and probable incompleteness of LC data across different clinical pathways. A broader issue is also raised in terms of the general population of those with LC who are referred to clinics but do not subsequently attend. The lack of data for LC clinics prevented further demographic analysis of these groups. Feedback from our LC clinic leads, however, suggested this pattern of under‐referral particularly impacted those from disadvantaged groups, and our qualitative data highlighted problems with access to specialist care for these populations. The findings from primary care data indicate a lack of overall alignment of data sources across the LC care pathway, which limits opportunities to understand the equity of provision for LC healthcare using administrative health record data alone.

### 3.6 What Good Practice Exists?

We found examples of inclusive practice from some clinics that had self‐assessed for inequities in access and were actively seeking to address these (Box 2). Two case studies (Boxes 3 and 4) provide an overview of inclusive approaches used within clinics adopting a strategic approach. Although formal evaluation of these initiatives by clinics was limited, there is considerable existing evidence that the approaches described can help reduce inequalities in healthcare and other public services [13].

Box 2 **Good practice initiatives in LC healthcare.**

Making inequities visible

- **Gathering and analysing local data** on clinic populations to inform initiatives that target under‐represented groups and areas (Leeds—see Box 3 case study for further details)
- **Monitoring outcomes** across different groups of people with LC to identify inequities in referrals and health outcomes (Leeds)

Improving referral to specialist care

Feedback from clinics suggested that the largest barrier to inclusive practice was reliance solely on GPs for referral. Referral processes were expanded in a number of ways to overcome this:

- **A decision‐making protocol and triage system** to improve collaboration and reduce inconsistencies between practitioners in the LC care pathway. This has led to better representation of different demographic groups in the LC clinic (NW London—see Box 4 case study)
- **Working with third sector partners** to improve understanding of LC and encourage presentation of symptoms at primary care (Leeds, Birmingham, Salford and Wales):
  ○ Designing a third sector referral system to enable referral recommendations directly from community groups rather than only via the GP gateway (Leeds)
  ○ Working with a Gypsy and Traveller advocacy group to plan social media and face‐to‐face information sessions and visits. Oversight of activity by a local authority network for minority ethnic communities (Leeds).

- **Working with GPs** to raise awareness about groups underrepresented in referrals for specialist care (Leeds, NW London); adding LC to the annual learning disability checklist and having a GP link worker supporting the local pathway supported uptake and information flow (Leeds)

Making LC services accessible

- **Offering multiple clinical sites** across urban centres (Leeds, NW London and Birmingham) to reduce travel time and financial burden.
- **Offering home visits, face‐to‐face appointments** and language support to tailor services to the needs of people with LC (Leeds).
- **Tackling Digital Literacy**—offering telephone assessments to people with LC who do not wish to use the C19‐YRS app to complete their assessments (Leeds and Salford).
- **Offering both virtual and face‐to‐face** peer support services to people living with LC (Birmingham, Leeds and NW London)

Co‐ordinated care

- **An integrated LC care pathway** has been developed to improve referrals and access by linking the community health teams with LC clinical teams (NW London—see Box 4 case study).

Box 3 **Case study 1—leeds LC community rehabilitation service.**

**Using the data**: A deep dive into referral data aligned the postcodes of people with LC with indices of multiple deprivation (IMD) deciles. This highlighted that those from more deprived areas were under‐represented by 13%, while the least deprived deciles were over‐represented by 13%. A targeted approach to supporting GP referrals in more deprived areas has closed this gap to 4%. In contrast with most other LOCOMOTION LC clinics, referrals for ethnic minority groups are currently over‐represented compared to local population data. Targeted strategies to include these groups have increased the number of ethnic minority referrals, and current numbers may be a more accurate reflection of LC in these communities, which are known to have had higher rates of Covid‐19.

**Outreach and signposting:** To address inequalities in referral rates for deprived communities, homeless people, refugees and those from Gypsy Roma and Traveller (GTR) communities, clinic staff have reached out to specific GP practices that work with these populations. A third sector referral form to support direct referral to the service is being piloted. Working with a local GTR advocacy group has also led to the production of a social media video. Health promotion activities have been organised at drop‐in centres used by GTR communities as well as homeless people, refugees and asylum seekers.

A leaflet to raise awareness of LC has been distributed to third sector, health and local authority groups, including a minority ethnic network, and translated into the seven most common languages in Leeds. The network provides oversight of activities to engage minority ethnic communities. An easy‐read booklet with LC information has been designed for people with learning disabilities and presented to GPs. Referral criteria have been reduced from 12 to 6 weeks for people with learning disabilities to address the absence of any people with LC from this group. Posters highlighting the similarity between symptoms of ageing and those of LC have been targeted at care homes and neighbourhood health teams.

Digital exclusion is addressed through training for clinic staff and by signposting individuals to support for accessing equipment and improving digital literacy. Standard pathways are adapted to accommodate those who lack technology skills or require additional assistance.

**Monitoring Outcomes:** Measures such as EQ5D (a standardised measure to record quality of life) are used to monitor treatment outcomes for any inequalities between diverse groups of people with LC. Comparison of these outcomes for those from minority ethnic backgrounds and those from the most deprived backgrounds against ‘all people with LC’ has shown no significant differences in quality of life.

Box 4 **Case study 2—NW london post‐covid integrated care board.**

NW London Post‐Covid Integrated Care Board included three secondary care clinic providers and six LC community teams covering eight boroughs. Collaboration between these teams over approximately 2–3 years aimed to overcome some of the barriers to support identified by people with LC and specialist clinics

**An integrated pathway** has been developed to improve consistency in referrals and access across the area by linking the community multidisciplinary teams (MDTs) with the acute teams. Patients are referred to a central Single Point of Access, hosted by one of the community services with GP support. This means that all referrals are triaged (i.e., sorted according to symptoms presented and clinical needs) and sent to either:

1. A Post‐Covid Assessment Clinic (PCAC) which is secondary care‐based and delivered predominantly face‐to‐face, with a respiratory or infectious diseases consultant, physiotherapist, psychologist and occupational therapist or
2. An LC Community team—Allied Health Professional rehabilitation services with a GP lead for support. Community teams are supported by the PCAC

**Triage of referrals with a protocol to support decision‐making** is supported by all Community MDTs across NW London. This has led to better representation of different demographic groups within the LC clinic. A data dashboard has been used to help understand where there is a need to reach out to under‐represented groups and develop initiatives. For example, GP practices that have less than 5% referrals are being targeted by LC teams, using an animation video to educate GPs and encourage referrals [34]

## 4 Discussion

### 4.1 Summary

A growing body of evidence highlights health inequalities in the prevalence and experience of LC in socially excluded groups, but evidence about practice initiatives to reduce inequalities is lacking. Our findings triangulate data from diverse sources to provide insight into patterns of LC diagnosis and referral in primary care, along with practical initiatives to address inequalities in access to specialist healthcare. We provide initial evidence that these initiatives could improve access for deprived and minority ethnic groups, but there is a substantial need for more detailed evaluation.

### 4.2 Strengths and Limitations

Involvement of people with LC in the research design and evaluation of LC services is a key strength of the study. Our mixed‐methods approach brings together multiple data sources to triangulate and explore diverse perspectives on LC inequalities.

Quantitative data collection and analysis were informed by qualitative findings; however, this analysis was limited by the incomplete and patchy data on excluded groups from clinical pathways. In primary care, the small number of people from specific disadvantaged groups was a further barrier to analysing inequities between population groups. We were unable to recruit homeless people, but gained some insights into why LC is difficult to identify in this group of people with LC. As an exploratory study, we had very limited evidence of the success of inclusion initiatives, and further research to evaluate these interventions is needed.

### 4.3 Comparison With Existing Literature

Accessing healthcare support for LC is reported as a problem for many people with LC [16, 26]; however, our findings reflect the additional disadvantage for socially excluded groups highlighted in the wider literature on Covid‐19: being accorded low credibility by healthcare practitioners, inadequate information about LC and available support, and mistrust of the healthcare system [27, 28].

Some LC studies have utilised the concept of epistemic injustice to better understand the process through which individuals' explanations of their symptoms are ignored and the harms caused by misrecognition [15, 29, 30]. Such injustice includes a racialised and gendered element for minority ethnic and female people with LC, who can feel both ignored in terms of inadequate information and care and also worried that they are being neglected because of their ethnic background or sex. This uncertainty is an important consideration for ethnic minorities and women navigating healthcare, creating doubt about the basis on which healthcare professionals respond or provide treatment [31]. KIs described how discrimination and inadequate information were linked to systemic injustice, mistrust and under‐representation in LC specialist care. This dimension of social exclusion goes beyond the usual power imbalance between those receiving care and healthcare practitioners, adding concerns about how much service users can trust practitioners [15]. Involving community advocacy groups in the referral pathway for LC care seems a promising good practice initiative, given that expertise in engaging marginalised groups is most likely to be found in third sector organisations [17, 32].

Failure to refer from primary care to specialist support is a further key issue for people with LC from underserved groups, exacerbating the potential mistrust in practitioners and creating further downstream consequences for health‐seeking behaviour in these populations [15, 28]. Our findings on barriers to LC support may help explain the lower rate of LC diagnosis found in primary care records for the most deprived quintile [25]. The lack of overlap between primary care and LC clinic data aligns with other recent work highlighting the probable under‐reporting of LC diagnosis in primary care, particularly for minority ethnic groups [33].

The good practice initiative to improve consistency in LC healthcare decision‐making (Box 4) is likely to be helpful in addressing the higher prevalence of LC among deprived and some minority ethnic populations found in studies with large samples [4, 25, 35]. One study suggests that a focus on symptoms rather than self‐report could better expose inequalities [4], and this approach could potentially add to evidence that LC symptoms may differ between ethnic groups [36, 37].

Being able to access support for LC involves navigating the healthcare system; this requires high levels of social capital and what Janet Shim [38] has termed ‘cultural health capital’. Shim defined this as:

‘the repertoire of cultural skills, verbal and nonverbal competencies, attitudes and behaviours, and interactional styles, cultivated by patients and clinicians alike, that, when deployed, may result in more optimal health care relationships. I consider cultural health capital alongside existing frameworks for understanding clinical interactions, and I argue that the concept of cultural health capital offers theoretical traction to help account for several dynamics of unequal treatment’.

(Shim, 2010: 1)

This definition is useful for better understanding the experiences of underserved communities living with LC, not least because it describes the kind of confidence, attributes and networks needed to navigate healthcare encounters with clinicians to receive appropriate care [15]. It also highlights the role of the clinician in shaping equitable encounters. If practitioners deploy attitudes and behaviours that discriminate rather than ensure optimal care, the encounter could reduce the confidence of health service users and negatively affect their interactions, restricting their cultural health capital in the process.

### 1 Implications for Research and/or Practice

Our triangulated findings, which include the perspective of those least likely to shape healthcare services, demonstrate that access to specialist support is a key issue for equitable provision in LC healthcare. The higher likelihood of being diagnosed with LC for minority ethnic and deprived people with LC does not translate to higher rates of referral to specialist LC care, according to those with lived experience of LC and clinic staff in our sample. A single point of entry to LC care pathways via GPs was, consequently, inadequate, and involving community advocacy groups in referrals showed positive impacts on access.

The use of a protocol focused on symptoms reported by people with LC to aid referral decisions is also promising in terms of existing research as well as our qualitative findings [39]. This approach reduces practitioner inconsistency and accords with effective methods for removing discriminatory practice which may affect excluded populations [13] as well as reducing an over‐reliance on self‐report. Our findings suggest that self‐report is less likely for populations that experience mistrust in healthcare providers and barriers to accessing knowledge and information about LC symptoms.

Evaluation of inclusion initiatives requires good‐quality data and resources for analysis [12]. Data quality, particularly in LC specialist care, is currently a stumbling block to assessing health outcomes, as there is no way of linking data from specialist centres to Electronic Health Records. The low numbers of people with an LC diagnosis being referred to specialist clinics, and even lower numbers attending appointments, make it even more difficult to assess the effectiveness of access initiatives by specialist centres. The paucity of qualitative or clinical data on homeless people with LC further highlights the invisibility of this population and signals an urgent need to identify such individuals and specifically target their care.

## 5 Conclusions and Recommendations

A key recommendation arising from our research is increased collaboration between LC clinics, third sector organisations and GPs to increase awareness and validation of LC, trust in healthcare and consistency of access to LC support, all of which could improve access to specialist LC care. Such initiatives are particularly important for those with low levels of health literacy and least capacity to persevere with requests for support.

In terms of care pathways, evidence from qualitative interviews and clinical data highlights the fragmented nature of the care people with LC could receive. This suggests that the integrated and multidisciplinary care pathway operated in NW London is an appropriate approach to diagnosis, referral and treatment of LC. Qualitative findings suggest that such collaboration across clinical teams could particularly benefit those who experience a range of symptoms, particularly if these fluctuate over time.

Some of the inclusive practice initiatives we identified within LC clinics such as Leeds are currently promoted within NHS LC policy [18]; however, our findings suggest there is a need for more implementation support, as these initiatives were not often utilised in practice. The LC policy should also more clearly promote collaboration between diverse specialities and sectors *within* the NHS as well as work to create links across sectors.

## Author Contributions

**Ghazala Mir:** conceptualisation, methodology, data curation, formal analysis, supervision, funding acquisition, writing – original draft, writing – review and editing, project administration, visualisation. **Jordan Mullard:** investigation, validation, formal analysis, project administration, writing – original draft, writing – review and editing, visualisation. **Amy Parkin:** writing – review and editing, investigation. **Cassie Lee:** writing – review and editing, investigation, validation, formal analysis. **Jonathan Clarke:** data curation, formal analysis, investigation project administration, writing – review and editing, writing – original draft, funding acquisition, methodology, visualisation. **Johannes H. De Kock:** writing – review and editing. **Denys Prociuk:** formal analysis, writing – original draft, writing – review and editing, data curation, investigation, visualisation. **Julie L. Darbyshire:** writing – review and editing. **Sophie Evans:** writing – review and editing, supervision, validation. **Manoj Sivan:** conceptualisation, methodology, data curation, supervision, funding acquisition, project administration, writing – review and editing.

## Disclosure

The views expressed in this publication are those of the authors and not necessarily those of NIHR or the Department of Health and Social Care.

## Ethics Statement

Bradford Leeds Research Ethics Committee (reference: 21/YH/0276) and the Health Research Authority Confidentiality Advisory Group under reference: 303623.

## Consent

Patient consent was obtained for all qualitative data interviews. All necessary permissions were obtained from the HRA and Whole Systems Integrated Care Data Access Group for anonymised primary care data use.

## Conflicts of Interest

The authors declare no conflicts of interest.

## Supporting information

Supp material 1‐ LC Interview Schedule.

Supp material 2 ‐ KI Interview Schedule.

Supp material 3 ‐ LC clinic survey.

## Data Availability

Aggregate datasets used for this publication are available upon reasonable request from the LOCOMOTION Chief Investigator, Professor Manoj Sivan.

## Endnotes

1 Supplementary material: Interview schedule for people with LC.

2 Supplementary material: Interview schedule for KIs.

3 Supplementary material: LC clinic survey questions.

## References

[1] National Institute for Health and Care Excellence (NICE) . Clinical Knowledge Summaries—Long Term Effects of Coronavirus [Long COVID] 2022, accessed July 6, 2024, https://cks.nice.org.uk/topics/long-term-effects-of-coronavirus-long-covid/background-information/definition/.

[2] J. B. Soriano , S. Murthy , J. C. Marshall , P. Relan , and J. V. Diaz , “A Clinical Case Definition of Post‐COVID‐19 Condition by a Delphi Consensus,” Lancet Infectious Diseases 22, no. 4 (2022): e102–e07, 10.1016/s1473-3099(21)00703-9. 34951953 PMC8691845

[3] Office For National Statistics (ONS) . Prevalence of Ongoing Symptoms Following Coronavirus (COVID‐19) Infection in the UK: 30 March 2023, accessed February 26, 2024, https://www.ons.gov.uk/peoplepopulationandcommunity/healthandsocialcare/conditionsanddiseases/bulletins/prevalenceofongoingsymptomsfollowingcoronaviruscovid19infectionintheuk/30march2023.

[4] A. Subramanian , K. Nirantharakumar , S. Hughes , et al., “Symptoms and Risk Factors for Long Covid in Non‐Hospitalized Adults,” Nature Medicine 28, no. 8 (2022): 1706–1714, 10.1038/s41591-022-01909-w. PMC9388369 35879616

[5] J. Kwon , R. Milne , C. Rayner , et al., “Impact of Long COVID on Productivity and Informal Caregiving,” European Journal of Health Economics, no. 7 (2023): 1095–1115, https://pubmed.ncbi.nlm.nih.gov/38146040/. 10.1007/s10198-023-01653-z PMC11377524 38146040

[6] C. Chen , S. R. Haupert , L. Zimmermann , X. Shi , L. G. Fritsche , and B. Mukherjee , “Global Prevalence of Post‐Coronavirus Disease 2019 (COVID‐19) Condition or Long Covid: A Meta‐Analysis and Systematic Review,” Journal of Infectious Diseases 226, no. 9 (November 1, 2022): 1593–1607. 35429399 10.1093/infdis/jiac136 PMC9047189

[7] L. L. O'Mahoney , A. Routen , C. Gillies , et al., “The Prevalence and Long‐Term Health Effects of Long Covid Among Hospitalised and Non‐Hospitalised Populations: A Systematic Review and Meta‐Analysis,” EClinicalMedicine 55 (2023), https://www.thelancet.com/journals/eclinm/article/PIIS2589-5370(22)00491-6/fulltext. 10.1016/j.eclinm.2022.101762 PMC9714474 36474804

[8] R. J. O'Connor , A. Parkin , G. Mir , et al., “Work and Vocational Rehabilitation for People Living With Long Covid,” BMJ 385 (2024): 385. 10.1136/bmj-2023-076508 38729647

[9] D. Ayoubkhani , F. Zaccardi , K. B. Pouwels , et al., “Employment Outcomes of People With Long Covid Symptoms: Community‐Based Cohort Study,” European Journal of Public Health 34, no. 3 (2024): 489–496, 10.1093/eurpub/ckae034. 38423541 PMC11161149

[10] K. L. Bentley‐Edwards , O. Adisa , K. E. Ruff , E. S. McClure , and W. R. Robinson , “Race, Racism, and Covid‐19 in the US: Lessons Not Learnt,” BMJ 384 (2024): e076106, 10.1136/bmj-2023-076106. 38408791

[11] E. Ladds , A. Rushforth , S. Wieringa , et al., “Persistent Symptoms After Covid‐19: Qualitative Study of 114 ‘Long Covid’ Patients and Draft Quality Principles for Services,” BMC Health Services Research 20, no. 1 (2020): 1144, 10.1186/s12913-020-06001-y. 33342437 PMC7750006

[12] G. Mir , N. Durrani , R. Julian , Y. Kimei , S. Mashreky , and T. T. D. Doan , “Social Inclusion and Sustainable Development: Findings From Seven African and Asian Contexts,” Sustainability 16, no. 11 (2024): 4859.

[13] G. Mir , S. Karlsen , W. V. Mitullah , et al., 2020. Achieving SDG 10: A Global Review of Public Service Inclusion Strategies for Ethnic and Religious Minorities. UNRISD Occasional Paper‐Overcoming Inequalities in a Fractured World.

[14] United Nations (UN). World Social Situation 2016: Leaving No One Behind—The Imperative of Inclusive Development 2016, accessed September 3, 2024, https://www.un.org/development/desa/socialperspectiveondevelopment/2016/09/06/world-social-situation-2016-leaving-no-one-behind-the-imperative-of-inclusive-development/#:~:text=A%20central%20pledge%20of%20the%202030%20Agenda%20is,to%20endeavour%20to%20reach%20the%20furthest%20behind%20first.

[15] J. Mullard , G. Mir , C. Herbert , et al., “‘You're Just a Guinea Pig’: Exploring the Barriers and Impacts of Living With Long COVID‐19: A View From the Undiagnosed,” Sociology of Health & Illness 46, no. 8 (2024): 1602–1625, 10.1111/1467-9566.13795. 38850204

[16] S. A. Baz , C. Fang , J. D. Carpentieri , and L. Sheard , “‘I Don't Know What to Do or Where to Go’. Experiences of Accessing Healthcare Support From the Perspectives of People Living With Long Covid and Healthcare Professionals: A Qualitative Study in Bradford, UK,” Health Expectations 26, no. 1 (2023): 542–554. 36512382 10.1111/hex.13687 PMC10124541

[17] J. Mullard , J. Kawalek , A. Parkin , et al., “Towards Evidence‐Based and Inclusive Models of Peer Support for Long Covid: A Hermeneutic Systematic Review,” Social Science & Medicine 320 (2023): 115669. 36708608 10.1016/j.socscimed.2023.115669 PMC9840228

[18] National Health Service (NHS) . Long COVID: The NHS Plan for 2021/22, accessed September 3, 2024, https://www.england.nhs.uk/coronavirus/publication/long-covid-the-nhs-plan-for-2021-22/.

[19] M. Sivan , T. Greenhalgh , J. L. Darbyshire , et al., “LOng COvid Multidisciplinary Consortium Optimising Treatments and Services AcrOss the NHS (LOCOMOTION): Protocol for a Mixed‐Methods Study in the UK,” BMJ Open 12, no. 5 (2022): e063505. 10.1136/bmjopen-2022-063505 PMC9114312 35580970

[20] National Institute for Health and Care Research (NIHR) . Improving Inclusion of Under‐Served Groups in Clinical Research: Guidance From INCLUDE Project. 2020, accessed January 14, 2025, www.nihr.ac.uk/documents/improving-inclusion-of-under-served-groups-in-clinical-research-guidance-from-include-project/25435.

[21] J. Ritchie and L. Spencer , “Qualitative Data Analysis for Applied Policy Research.” Analyzing Qualitative Data (Routledge, 2002), 173–194.

[22] T. Greenhalgh , J. L. Darbyshire , C. Lee , E. Ladds , and J. Ceolta‐Smith , “What Is Quality in Long Covid Care? Lessons From a National Quality Improvement Collaborative and Multi‐Site Ethnography,” BMC Medicine 22, no. 1 (2024): 159, 10.1186/s12916-024-03371-6. 38616276 PMC11017565

[23] L. A. Curry , H. M. Krumholz , A. O'cathain , V. L. Plano Clark , E. Cherlin , and E. H. Bradley , “Mixed Methods in Biomedical and Health Services Research,” Circulation. Cardiovascular Quality and Outcomes 6, no. 1 (January 2013): 119–123. 23322807 10.1161/CIRCOUTCOMES.112.967885 PMC3569711

[24] T. H. Liu , P. Y. Huang , J. Y. Wu , et al., “Post‐COVID‐19 Condition Risk in Patients With Intellectual and Developmental Disabilities: A Retrospective Cohort Study Involving 36,308 Patients,” BMC Medicine 21, no. 1 (December 2023): 505. 38114989 10.1186/s12916-023-03216-8 PMC10731815

[25] D. Prociuk , J. Clarke , N. Smith , et al., LOCOMOTION Consortium, “Understanding the Clinical Characteristics and Timeliness of Diagnosis for Patients Diagnosed With Long COVID: A Retrospective Observational Cohort Study From North West London,” *medRxiv*, August, 2024. 10.1111/hex.70429 PMC12462644 40996585

[26] Healthtalk.org Seeking Help From the GP. The Dipex Charity 2024, accessed 9 September 2024, https://healthtalk.org/experiences/long-covid-in-adults/seeking-help-gp/.

[27] J. Nazroo , K. Murray , H. Taylor , et al., Rapid Evidence Review: Inequalities in Relation to COVID‐19 and Their Effects on London (University of Manchester, The Ubele Initiative, University of Sussex, 2020).

[28] K. Fenton , E. Pawson , and L. de Souza‐Thomas , Beyond the Data: Understanding the Impact of COVID‐19 on BAME Groups (Public Health England, 2020).

[29] M. Fricker , Epistemic Injustice: Power and the Ethics of Knowing (Oxford University Press, 2007).

[30] J. Ireson , A. Taylor , E. Richardson , et al., “Exploring Invisibility and Epistemic Injustice in Long Covid—A Citizen Science Qualitative Analysis of Patient Stories From an Online Covid Community,” Health Expectations 25, no. 4 (2022): 1753–1765. 35557480 10.1111/hex.13518 PMC9327841

[31] K. Craig‐Henderson , Navigating the Inequitable US Healthcare System: In Search of Critical Care (Anthem Press, 2024).

[32] G. Mir and A. Sheikh , “‘Fasting and Prayer Don't Concern the Doctors… They Don't Even Know What It Is’: Communication, Decision‐Making and Perceived Social Relations of Pakistani Muslim Patients With Long‐Term Illnesses,” Ethnicity & Health 15, no. 4 (2010): 327–342. 20544446 10.1080/13557851003624273

[33] A. Knuppel , A. Boyd , J. Macleod , N. Chaturvedi , and D. M. Williams , “The Long COVID Evidence Gap in England,” Lancet 403, no. 10440 (May 18, 2024): 1981–1982. 38729195 10.1016/S0140-6736(24)00744-X

[34] North‐West London . Long Covid Animation for Professionals: NHS North West London, 2024.

[35] S. Shabnam , C. Razieh , H. Dambha‐Miller , et al., “Socioeconomic Inequalities of Long COVID: A Retrospective Population‐Based Cohort Study in the United Kingdom,” Journal of the Royal Society of Medicine 116, no. 8 (2023): 263–273. 37164035 10.1177/01410768231168377 PMC10469969

[36] D. Khullar , Y. Zhang , C. Zang , et al., “Racial/Ethnic Disparities in Post‐Acute Sequelae of SARS‐CoV‐2 Infection in New York: An EHR‐Based Cohort Study From the RECOVER Program,” Journal of General Internal Medicine 38, no. 5 (April 2023): 1127–1136. 36795327 10.1007/s11606-022-07997-1 PMC9933823

[37] M. M. Jacobs , E. Evans , and C. Ellis , “Racial, Ethnic, and Sex Disparities in the Incidence and Cognitive Symptomology of Long COVID‐19,” Journal of the National Medical Association 115, no. 2 (April 2023): 233–243. 36792456 10.1016/j.jnma.2023.01.016 PMC9923441

[38] J. K. Shim , “Cultural Health Capital: A Theoretical Approach to Understanding Health Care Interactions and the Dynamics of Unequal Treatment,” Journal of Health and Social Behavior 51, no. 1 (2010): 1–15, 10.1177/0022146509361185. 20420291 PMC10658877

[39] M. Sivan , S. Halpin , L. Hollingworth , N. Snook , K. Hickman , and I. J. Clifton , “Development of an Integrated Rehabilitation Pathway for Individuals Recovering From COVID‐19 in the Community,” Journal of Rehabilitation Medicine 52, no. 8 (August 2020): jrm00089. 32830284 10.2340/16501977-2727

