# Supplementary material for: Addressing Inequalities in Long Covid Healthcare: A Mixed‐Methods Study on Building Inclusive Services
Source: Health Expect. 2025 Jul 2;28(4):e70336. doi: 10.1111/hex.70336 (PMC12215820; doi:10.1111/hex.70336)
Supplement: Supplementary file 1 — Supp material 1‐ LC Interview Schedule. [file HEX-28-e70336-s002.docx]

# PATIENT PARTICIPANT INTERVIEW SCHEDULE

**Study Title: Understanding inequalities in Long Covid and in use of Long Covid services**

*We'd like to invite you to take part in our research study. Before you decide, it is important that you understand why the research is being done and what it would involve for you. Please take time to read this information and discuss it with others if you wish. If there is anything that is not clear, or if you would like more information, please ask us.*

## What is the purpose of the study?

Our research aims to produce the best care possible for people with Long Covid by looking at what happens when they receive support from health services across the country. We will create new systems of care and support for people with Long Covid, and check how these are working to create the best care we can.

We want to understand whether certain groups of people are less able to access or are not receiving the appropriate support and care, such as those with less income or people who face discrimination in society. Importantly, we hope to make recommendations for positive change so everyone can easily get the support they need, regardless of where they live or their background.

## Why have I been invited?

You have been invited to take part in this research study because you have had continuing illness months after you were infected with COVID-19.

## Do I have to take part?

No, taking part in this study is entirely voluntary and you can withdraw at any time if you later change your mind, without giving a reason.

## What will happen to me if I decide to take part?

We will invite you to be interviewed for the study at a place of your choice, that can be online, via telephone or in person. If you would like support using MS Teams or Zoom, we can provide you with an online tutorial video and 1:1 help is also available from the research team, should you need it.

If you require a translator present for your interview, please let us know as this can be provided.

The interview will be for up to an hour and will be audio or video recorded so that we do not miss any details of what you tell us. You can do the interview with a relative or carer present if you want, and if you think you’ll get tired we can break them up the interview into shorter sessions of 15-30 minutes, done on different days. We are aware that the interview might cause unpleasant memories from your illness to surface, or cause you to focus on your current difficulties. We will pause or stop the interview should you become upset or distressed. We can also refer for further support if you feel this would be helpful.

Questions will cover your experience of COVID-19, including:

- your symptoms
- any help you tried to find in order to get better and who supported you
- your use of healthcare services and attitudes towards healthcare support
- Other workstream overlap questions
- your views about Long Covid
- any negative reactions you have had from others
- any things about your experience that made you feel angry, frustrated or emotional
- Can you tell me a little bit of background about when you first had Covid and the symptoms you had as a result?
- How would you describe your health before getting Covid?
- Did you take a Lateral flow or a PCR test when you suspected you may have Covid? Why did you decide to do that? If not, what were the reasons for not requesting a test?
- What kind of support did you get when you caught the virus?
- Was there any other kind of support you needed or tried to get?
- What advice did you get from healthcare professionals? How helpful did you find this?
- Did healthcare staff ever mention Long Covid or specific help for this?
- Have you ever been to your GP for help with managing Long Covid? If so, how often? If not, what are the reasons for this?
- Did you find that there were any barriers to getting help? If so, what were they? [prompt if necessary: access issues, lack of time, childcare, registration with GP, lack of address etc]
- Do any particular activities, events, thoughts or feelings trigger your symptoms or make your symptoms worse?
- Exposure?
- Pre-existing medical conditions/disabilities?
- Is the area you live in regarded as deprived?
- Would you be interested in being part of our PPI group for the project?

A university-approved service will write out what is said in these meetings (this is called transcription). The audio files will be anonymised and the recordings will be destroyed at the end of the study.
