# Supplementary material for: Addressing Inequalities in Long Covid Healthcare: A Mixed‐Methods Study on Building Inclusive Services
Source: Health Expect. 2025 Jul 2;28(4):e70336. doi: 10.1111/hex.70336 (PMC12215820; doi:10.1111/hex.70336)
Supplement: Supplementary file 2 — Supp material 2 ‐ KI Interview Schedule. [file HEX-28-e70336-s003.docx]

**Understanding inequalities in Long Covid and in use of Long Covid services study**

**Interview Schedule:**indicative questions for KIs

(Semi-structured format)

**What is the purpose of the study?**

Our research aims to produce the best care possible for people with Long Covid by looking at what happens when they receive support from health services across the country. We will create new systems of care and support for people with Long Covid, and check how these are working to create the best care we can.

We want to understand whether certain groups of people are less able to access support and care, such as those with less income or people who face discrimination in society. Importantly, we hope to make recommendations for positive change so everyone can easily get the support they need, regardless of where they live or their background.

**Why have I been invited?**

You have been invited to take part in this research study because you have contact with people from marginalised groups who have been affected by COVID-19. This might be through your professional or voluntary work or because you are an expert in the field of Covid or Long Covid.

**Questions**

1. Can I ask in what ways you have been involved in Covid and/or Long Covid service provision or research?
2. What work have you done to explore the disproportionate impacts of Covid on marginalised communities?
3. Do you expect to see the same kind of disproportionality amongst those diagnosed with Long Covid?
4. What is your view on how Long Covid is affecting people from marginalised communities?
5. Can you explain a bit about your position on this (prompts – evidence from research findings or professional experience)?
6. Research evidence to date indicates that current rates of Long Covid are higher within particular demographic groups (white, middle-aged women, deprived populations and those with existing health conditions or more exposed to risk through their work). Do you think this is a true reflection of prevalence within the population? Please explain why you think so.
7. In terms of ethnicity, do you have any views about why the current evidence suggests non-white ethnicity is associated with a lower risk of LC despite the higher known risks for Covid-19? (Prompts: sample size, ethnic groupings)
8. What sorts of feedback about healthcare support are you getting from marginalised communities, and which specific communities is this from (prompts: diagnosis, access to support, referral, practitioner attitudes)?
9. Have you had any feedback about the impact of LC from people in these communities (prompts: work, finances, parenting, caring duties, emotional/mental health, relationships) ?
10. Are you aware of any particular triggers for LC symptoms that affect people from socially excluded groups?
11. What is your current knowledge or involvement in Long Covid Clinics?
12. Anecdotal evidence suggests that a lot of LC symptoms are being masked by previous ill health or are being misunderstood by people, which results in them not getting help. Do you think this is an issue for people from excluded populations?
13. Do you think that there is some misinformation or confusion around Long Covid symptoms that could be preventing people from accessing services? To what degree do you think this is a misunderstanding by patients or confusion over diagnosis in referring practitioners, or both?
14. What do you think can be done to mitigate this confusion and do you have any examples of best practice around diagnosis, treatment, or self-identification of Long Covid?
15. From your research or experience, why do you think some communities may be better at accessing LC services than others?
16. What kinds of barriers do you think would prevent LC services reaching marginalised communities?
17. Are there areas of provision that you feel may inadvertently exclude people from marginalised backgrounds for example, those on low incomes, people from different ethnic minorities, people with physical or cognitive disabilities?
18. What challenges do you think Clinics face in widening participation?
19. We’re currently exploring different forms of support that people with Long Covid may be using instead of clinical provision, have you heard of any cases or examples of different forms of support accessed by people from excluded groups?
20. Many people with Long Covid are out of work or have reduced their working days, to what extent is this an issue among marginalised populations?
21. ? I wonder if you could tell me what you think makes the difference in terms of work-based support or support to remain/return to work for people from socially excluded groups?
22. To conclude, I wonder if you have any examples or suggestions of best practice in supporting those with Long Covid and what kinds of services would work to meet the needs of people with this condition?
23. Do you think provision would need to be tailored in any way to meet the specific needs of underrepresented groups and if so, what do you think this would look like?

Are there any other experiences, issues or expert knowledge you’d like to share or provide that relates to support for people with Long Covid?
