# Supplementary material for: Addressing Inequalities in Long Covid Healthcare: A Mixed‐Methods Study on Building Inclusive Services
Source: Health Expect. 2025 Jul 2;28(4):e70336. doi: 10.1111/hex.70336 (PMC12215820; doi:10.1111/hex.70336)
Supplement: Supplementary file 3 — Supp material 3 ‐ LC clinic survey. [file HEX-28-e70336-s001.docx]

**Collecting and using data to inform inclusive practice in long covid services**

**Site:**

**What data:**

- Do you record the following? Yes/No
  - Age, Gender, Ethnicity, Social Deprivation, Disability (physical, mental, or intellectual) and Homelessness

**Accessing data:**

- Where is this data recorded, ie where can you access it from e.g. a Local Specific Long Covid system, a hospital-based system or an integrated primary care system?
- How is this recorded [i.e free text/codes]?

**How is this data used?**

- Do you evaluate your service using these metrics [yes/no]
  - If yes, explain how you use these metrics for evaluation [free text]
  - If no, are you doing any other work to evaluate or target your service for different demographic groups? [free text]
- If you are not currently collecting or evaluating data in relation to these characteristics, do you plan to? Please add any details you can of planned work.
- As a result of analysing the specific information have you undertaken any projects/changes? Please give details

**Experience of using data?**

- What helps or hinders collection/use of data on this characteristic (eg poor completion rates; confusion about requirements; disagreement with policy)?
